# MAP Kinase Cascades in *Arabidopsis* Innate Immunity

**DOI:** 10.3389/fpls.2012.00169

**Published:** 2012-07-24

**Authors:** Magnus W. Rasmussen, Milena Roux, Morten Petersen, John Mundy

**Affiliations:** Department of Biology, University of Copenhagen,Copenhagen, Denmark

**Keywords:** calcium signaling, hypersensitive response, MAP kinase cascade, MAP kinase substrates, pathogen effectors, pattern recognition receptors, reactive oxygen species, resistance proteins

## Abstract

Plant mitogen-activated protein kinase (MAPK) cascades generally transduce extracellular stimuli into cellular responses. These stimuli include the perception of pathogen-associated molecular patterns (PAMPs) by host transmembrane pattern recognition receptors which trigger MAPK-dependent innate immune responses. In the model *Arabidopsis*, molecular genetic evidence implicates a number of MAPK cascade components in PAMP signaling, and in responses to immunity-related phytohormones such as ethylene, jasmonate, and salicylate. In a few cases, cascade components have been directly linked to the transcription of target genes or to the regulation of phytohormone synthesis. Thus MAPKs are obvious targets for bacterial effector proteins and are likely guardees of resistance proteins, which mediate defense signaling in response to the action of effectors, or effector-triggered immunity. This mini-review discusses recent progress in this field with a focus on the *Arabidopsis* MAPKs MPK3, MPK4, MPK6, and MPK11 in their apparent pathways.

## INTRODUCTION

Plants have evolved an effective basal defense system to detect and limit the growth of pathogens. Pathogens may be recognized by the host via the perception of conserved microbial structures termed pathogen-associated molecular patterns (PAMPs). PAMPs are recognized via transmembrane pattern recognition receptors (PRRs) that bind specific PAMPs and initiate intracellular immune responses (Zipfel, 2008). These PAMP-triggered immunity (PTI) responses include the generation of reactive oxygen species (ROS), extracellular alkalinization, and protein phosphorylation with associated gene regulation that ultimately restricts the growth of the microbial intruder (Gimenez-Ibanez and Rathjen, 2010).

**FIGURE 1:**
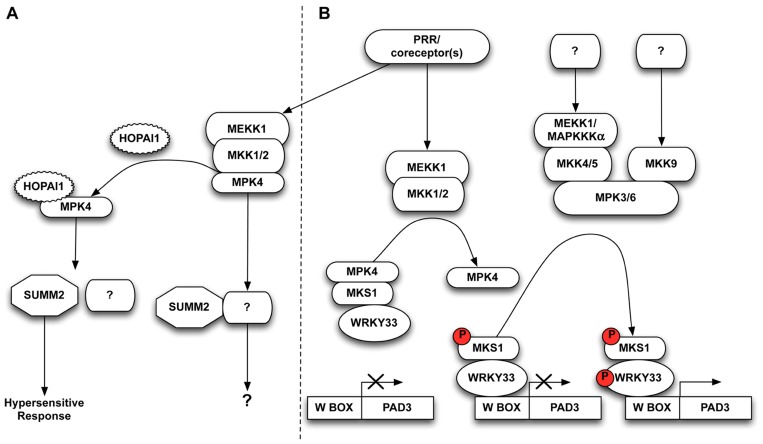
**(A)** MAPK signaling cascades are attractive targets for bacterial effectors. The *P. syringae* HopAI1 effector irreversibly inactivates MPK4 to prevent immune responses. The R protein SUMM2 may guard processes downstream of MPK4 independent from MKS1, and triggers a hypersensitive response in the event of loss or inactivation of MPK4. **(B)** PAMP perception by PRRs instigates a signaling cascade, often via co-receptors, which causes activation of MAP3K MEKK1 and two MAP2Ks MKK1 and MKK2. These phosphorylate and activate MPK4 which then phosphorylates its substrate MKS1, releasing MKS1 in complex with WRKY33. MPK3/MPK6 sequentially phosphorylate WRKY33 allowing it to promote *PAD3* transcription, thus activating plant defense.

Mitogen-activated protein kinase (MAPK) signaling plays central roles in such intracellular immunity pathways. In general, MAP kinase signaling is initiated by the stimulus-triggered activation of a MAP kinase kinase kinase (MAP3K; also called MEKK). MAP3K activation, which may be directly or indirectly effected by a PRR, in turn leads to the phosphorylation and activation of downstream MAP kinase kinases (MAP2K; also called MKK or MEK). Subsequently, the MAP2K phosphorylates the downstream MAPK sequentially leading to changes in its subcellular localization and/or phosphorylation of downstream substrates including transcription factors which alter patterns of gene expression (see **Figure 1**). General functions of MAPK cascades in plant biology have recently been reviewed elsewhere (Fiil et al., 2009; Rodriguez et al., 2010; Komis et al., 2011).

## MAPK CASCADES IN PTI

A few PRRs have been documented to stimulate MAPK signaling upon perception of PAMPs. These include the flagellin receptor FLS2 (Felix et al., 1999; Gómez-Gómez and Boller, 2000), the bacterial elongation factor EF-Tu receptor EFR (Zipfel et al., 2006), and the chitin receptor CERK1 (Miya et al., 2007).

The *Arabidopsis* genome encodes 60 MAP3Ks, 10 MAP2Ks, and 20 MAPKs (Ichimura et al., 2002). This indicates that MAPK cascades may not simply consist of single MAP3Ks, MAP2Ks, and MAPKs connected together. Instead, it suggests that there is some level of redundancy, and that the spatial and temporal activities of different components may be strictly regulated to minimize wanton cross-talk. The three MAPKs MPK3, MPK4, and MPK6 are the most intensively studied plant MAPKs, and all three were implicated in defense signaling a decade ago (Petersen et al., 2000; Asai et al., 2002). MPK11, a close homolog to MPK4, has also recently been shown to be activated by PAMP treatment (Bethke et al., 2012).

MPK3, MPK4, and MPK6 are all activated by PAMPs such as flg22 (a conserved 22 amino acid flagellin peptide) and elf18 (elongation factor-Tu peptide; Felix et al., 1999; Zipfel et al., 2006). However, these three MAPK cascades are differently regulated already at the PRR level. For example, the two receptor kinases BAK1 and BKK1 genetically regulate PAMP signaling through their interactions with cognate PRRs (Roux et al., 2011; Schwessinger et al., 2011). The BAK1 mutant allele *bak1-5* carries a Cys408Tyr substitution adjacent to its kinase catalytic loop. This impairs its flg22-regulated kinase activity and inhibits phosphorylation of MPK4. However, the catalytic complex formed between mutant BAK1 in *bak1-5* and FLS2 is still able to induce phosphorylation of MPK3/MPK6 (Roux et al., 2011; Schwessinger et al., 2011). Interestingly, MPK3/MPK6 phosphorylation was impaired in only the double *bak1-5 bkk1* background and not in the individual *bak1-5* and *bkk1* lines (Roux et al., 2011).

Asai et al. (2002) developed an elegant protoplast expression system in an attempt to identify signaling components downstream of FLS2. With this system they were able to show a complete MAPK cascade downstream of FLS2 consisting of the MAP3K MEKK1, two MAP2Ks (MKK4 and MKK5), and the MAPKs MPK3/MPK6. However, genetic evidence later showed that MEKK1 kinase activity was dispensable for MPK3/MPK6 activation, although *mekk1* plants were impaired in MPK4 activation (Rodriguez et al., 2007). Interestingly, expressing a kinase dead version of MEKK1 in *mekk1* plants completely restored the activation of MPK4 upon treatment with flg22, suggesting that MEKK1 may “simply” act as a scaffold protein (Rodriguez et al., 2007). Biochemical and genetic studies further revealed that the two MAP2Ks MKK1 and MKK2 interact with both MEKK1 and with MPK4, and that flg22-induced MPK4 activation is impaired in the double *mkk1 mkk2* mutant. This indicates that MKK1 and MKK2 are partially redundant in MPK4 mediated downstream signaling (Gao et al., 2008; Qiu et al., 2008b).

MPK4 was originally reported as a negative regulator of plant immunity because the *mpk4* mutant accumulates high levels of salicylic acid, constitutively expresses pathogenesis-related (*PR*) genes, and has a severely dwarfed growth phenotype (Petersen et al., 2000). This phenotype is very similar to that of the *mekk1* single and *mkk1 mkk2* double mutants, further supporting their functional relationships (Rodriguez et al., 2007; Gao et al., 2008; Qiu et al., 2008b).

## MAPK CASCADES IN EFFECTOR-TRIGGERED IMMUNITY

In addition to PTI, plants also employ resistance (R) proteins as cytoplasmic receptors to directly or indirectly recognize specific pathogenic effector proteins injected into host cells as virulence factors. Effector-triggered immunity (ETI) and PTI share a number of responses, although ETI also includes varying levels of rapid, localized cell death in what is called the hypersensitive response. R protein-dependent recognition initiates immune responses in ETI. R proteins may recognize effector proteins either directly or indirectly by monitoring changes in the effector’s host target(s). This latter case gave rise to the guard hypothesis in which R proteins guard host guardees that are manipulated by pathogen effectors (Van Der Biezen and Jones, 1998).

The genetic characterization of the MEKK1/MKK1–MKK2/ MPK4 cascade as a negative regulatory pathway of defense responses was at odds with the activation of the pathway by PAMPs. Instead, it was possible that the severe phenotypes of the kinase knockout mutants were caused by activation of one or more R protein(s) guarding this kinase pathway. Indeed, in an elegant screen for suppressors of the *mkk1 mkk2* double mutant, Zhang et al. (2012) identified the R protein SUMM2 (suppressor of *mkk1 mkk2*). The T-DNA insertion line *summ2-8* completely suppressed the severe *mkk1 mkk2* phenotype in respect to morphology, cell death, ROS levels and *PR* gene expression (Zhang et al., 2012). The analogous knockout phenotype of the upstream MAP3K *mekk1* is also completely suppressed in the *summ2-8* background. Interestingly, although the *mpk4* mutant shares a similar phenotype with the knockouts of its upstream kinase partners, the *mpk4* phenotype is not fully suppressed by the *summ2-8* mutation, as double *mpk4 summ2-8* mutants still retain residual cell death and low levels of ROS. This suggests that MPK4 is involved in other pathways independent of SUMM2, and that MPK4 may be guarded by additional R proteins (Zhang et al., 2012; **Figure 1A**).

The importance of MAPK signaling in immunity is emphasized by studies reporting bacterial effector proteins targeting MAPK cascades for downregulation (Zhang et al., 2007a,b, 2012; Cui et al., 2010). For example, the *Pseudomonas syringae* effector protein HopAI1 targets and irreversibly inactivates MPK3, MPK4, and MPK6, thereby suppressing immune responses which would otherwise inhibit bacterial growth (Zhang et al., 2007a, 2012). In addition, the *P. syringae* effector protein AvrB has been shown to interact with and induce the phosphorylation of MPK4, although it has not been shown if this phosphorylation occurs as a direct effect of AvrB action or via recognition of AvrB by the plant immune system (Cui et al., 2010).

In plants carrying functional SUMM2 alleles, immune responses are activated by bacterial effector proteins targeting the MPK4 pathway (**Figure 1A**). For example, inducible expression of the bacterial HopAI1 effector in wild-type plants gives rise to a defense phenotype similar to that seen in *mekk1*, *mkk1 mkk2*, and *mpk4* mutants including elevated levels of ROS, *PR* gene expression, and cell death (Zhang et al., 2012). SUMM2 apparently does not interact directly with the kinase components of the MEKK1/MKK1–MKK2/MPK4 signaling cascade, suggesting that SUMM2 most likely guards a downstream target of MPK4 activity (Zhang et al., 2012). At present, the best studied *in vivo* substrate of MPK4 activity is MPK4 substrate 1 (MKS1) which forms a nuclear complex with MPK4 and the WRKY33 transcription factor (Andreasson et al., 2005; Qiu et al., 2008a). Phosphorylation of MKS1 follows MPK4 activation by flg22 perception and, once phosphorylated, MKS1 is released from complexes with MPK4, thereby releasing the WRKY33 transcription factor to bind to its cognate target genes (Qiu et al., 2008a). It has therefore been proposed that MPK4 and MKS1 sequester WRKY33 in the absence of pathogens, and free WRKY33 to induce resistance upon pathogen perception (**Figure 1B**, left).

As MKS1 is the only known direct target of MPK4, Zhang et al. (2012) tested whether MKS1 interacted with the R protein SUMM2 that seemingly guards MPK4 activity. However, no interaction between SUMM2 and MKS1 was detected. Since *mks1* mutants have a wild-type growth phenotype, and the *mpk4* phenotype is strongly suppressed in the *mks1* background, SUMM2 may guard a process downstream of MPK4 that is independent of MKS1 (Petersen et al., 2010).

## WRKY TRANSCRIPTION FACTORS

The plant-specific WRKY family is a large group of transcription factors which bind a conserved W-box sequence in the promoters of numerous genes including those encoding PR proteins. WRKY33 was found to induce the transcription of *PHYTOALEXIN DEFICIENT 3* (*PAD3*) which encodes the cytochrome P450 monooxygenase 71B15 required for synthesis of the antimicrobial compound camalexin (Zhou et al., 1999; Qiu et al., 2008a; **Figure 1B**). The *wrky33* mutant exhibits enhanced susceptibility toward necrotrophic pathogens such as *Botrytis cinerea*, while WRKY33 overexpression results in increased resistance due to enhanced *PAD3* expression (Zheng et al., 2006).

MPK3 and MPK6 activities also induce the production of camalexin. Transient overexpression of the constitutively active, phospho-mimic mutant forms of MKK4/MKK5 (MKK4^DD^ and MKK5^DD^), which are the upstream MAP2Ks of MPK3/MPK6, has been reported to induce transcription of both *PAD2*, which encodes γ-glutamylcysteine synthetase functioning in glutathione biosynthesis, and *PAD3*. Both PAD2 and PAD3 are necessary for camalexin production (Parisy et al., 2007; Ren et al., 2008). Pathogen-induced camalexin accumulation is partially comprised in *mpk3* but not notably in *mpk6* mutants, yet camalexin accumulation in *mpk3 mpk6* double mutants is almost completely abolished (Ren et al., 2008). While this implicates MPK3/MPK6 in camalexin synthesis, caution should be applied in evaluating results obtained from the *mpk3 mpk6* double mutant as it is arrested at the cotyledon stage and is unable to initiate true leaves (Wang et al., 2007). Upstream of MPK3/MPK6 in camalexin induction, MKK4 and MKK5 are activated by the MAP3Ks MEKK1 and MAPKKKα in response to fungal pathogens (Ren et al., 2008). Yet another MAP2K, MKK9, whose upstream MAP3K(s) remains unidentified, is also involved in MPK3/MPK6 activation, as plants expressing phospho-mimic MKK9^DD^ produce even more camalexin than plants expressing MKK4^DD^ or MKK5^DD^ (Xu et al., 2008).

To delineate the link between MPK3/MPK6 activation and camalexin accumulation, Mao et al. (2011) elegantly introduced the phospho-mimic mutant *Nt*MEK2^DD^, an MKK4 and/or MKK5 ortholog from *Nicotiana tabacum*, into an array of different *wrky* mutants in a search for essential transcription factors involved in MPK3/MPK6 mediated camalexin induction. Interestingly, NtMEKK2^DD^ was able to induce camalexin accumulation in all tested mutant lines except *wrky33*. In addition, WRKY33 proved to be a substrate of MKP3/MPK6 activity, and overexpression of non-phosphorylatable forms of WRKY33 could not fully complement the inability of *wrky33* mutants to express *PAD3* and accumulate camalexin (Mao et al., 2011; **Figure 1B**, right).

WRKY33-induced *PAD3* expression therefore appears to involve both MPK4- and MPK3/MPK6-mediated signaling (Andreasson et al., 2005; Qiu et al., 2008a; Mao et al., 2011). Mao et al. (2011) proposed a model in which PAD3-mediated camalexin induction occurs differentially depending on the type of pathogen causing the immune response. In this model, bacterial pathogens induce an MPK4 mediated response while fungal pathogens initiate an MPK3/MPK6 mediated response. This hypothesis is based on overexpression of the constitutively active MKK4/MKK5 ortholog *NtMEKK2*^DD^, rendering MPK3/MPK6 hyperactive and able to induce *PAD3* expression (Mao et al., 2011). In support of this hypothesis, the *mpk3 mpk6* double mutant is comprised in *B. cinerea*-induced *PAD3* induction (Ren et al., 2008). Nonetheless, and as noted above, some care should be taken with experiments based on *mpk3 mpk6* double mutants given their developmental lethality (Wang et al., 2007).

An alternative model may therefore be proposed which combines the MPK4 and MPK3/MPK6 pathways into a dual control of *PAD3* regulation in response to pathogen perception (**Figure 1B**). In such a model, WRKY33 is sequestered in a nuclear complex comprising at least MPK4 and MKS1 in unchallenged plants, and is released following PAMP perception (Qiu et al., 2008a). Phosphorylation is dispensable for WRKY33 to bind its cognate W-box *cis*-elements, although it does promote transcriptional activation (Mao et al., 2011). This is illustrated by the fact that *PAD3* expression is induced in *mpk4* plants (Qiu et al., 2008a), perhaps due to the basal activity of free non-phosphorylated WRKY33 or by free WRKY33 activated by basal MPK3 and/or MPK6 activity. In this scenario, once WRKY33 is released from its nuclear complex with MPK4 and MKS1, it is phosphorylated and hence activated by MPK3/MPK6, thereby inducing camalexin levels through *PAD3* expression. The elevated *PAD3* expression induced from *NtMEKK2*^DD^ hyper-activated MPK3/MPK6 (Mao et al., 2011) is not in conflict with this model, as it is likely that hyperactive MPK3/MPK6 are able to phosphorylate residual free WRKY33, thus bypassing other possible feedback mechanisms in *PAD3* expression.

In this model, MPK4 and MPK3/MPK6 function together as a binary switch conferring dual level regulation. Clarification of the mode of action in which MPK4 and MPK3/MPK6 function clearly needs further elucidation and should include experiments using catalytically inactive and/or inactivatable MPK4 (Petersen et al., 2000; Brodersen et al., 2006). Application of fungal PAMPs to plants expressing catalytically inactive MPK4 might indicate whether phosphorylation of free WRKY33 by endogenous MPK3/MPK6 is enough to induce expression of *PAD3*.

## MAPK IN GENERAL STRESS SIGNALING

The refined work of Popescu et al. (2009) identified a MAP2K–MAPK phosphorylation network covering 570 MAPK substrates by combinatorially pairing active MAP2Ks with MAPKs, and then subjecting them to a protein microarray phosphorylation assay. Interestingly, the substrates identified were enriched for transcription factors involved in stress responses. Notably, MPK6 phosphorylated 32% of the identified targets, of which 40% overlapped with MPK3 targets (Popescu et al., 2009). This is in agreement with earlier data, similarly obtained from a protein microarray study (Feilner et al., 2005). Equally noteworthy is the finding that MPK3 also shared 50% of its targets with MPK4, revealing intensive synergy in MAPK signaling (Popescu et al., 2009).

In addition to MAPK cascades, ROS also play a pivotal role in stress signaling (Rodriguez et al., 2010). OXI1, a serine/threonine kinase induced by general ROS-generating stimuli, is required for full activation of MPK3/MPK6 after treatment with H_2_O_2_ (Rentel et al., 2004). Although OXI1 is characterized as an upstream regulator of MPK3/MPK6 activation, MPK3/MPK6 have been shown to phosphorylate OXI1 *in vitro*. This suggests that there is a feedback loop, but *in vivo* data supporting such a loop has not been shown (Forzani et al., 2011).

In addition to MAPK cascade signaling, PAMP perception also induces Ca^2+^ dependent kinases (CDPKs) by regulating Ca^2+^ influx channels (Ma et al., 2009; Kwaaitaal et al., 2011). Recent findings indicate that Ca^2+^ ATPases regulate Ca^2+^ efflux and function to regulate innate immune defenses (Zhu et al., 2010). Of particular interest is the Ca^2+^ ATPase ACA8 which was shown to interact with FLS2, and which may well regulate CDPK signaling through flg22 perception (Frei dit Frey et al., 2012).

MPK8 activity has been shown to negatively regulate the expression of *OXI1* in order to maintain ROS homeostasis. Remarkably, activation of MPK8 is not limited to the upstream MAP2K MKK3, as the Ca^2+^ binding protein calmodulin (CaM) is able to bind and activate MPK8 in an Ca^2+^-dependent manner (Takahashi et al., 2011). CaM-mediated MPK8 activation is interesting because it bypasses the traditional, sequential activation of MAPKs and also unequivocally links MAPK activation with the ROS burst and ion flux during stress signaling. In addition, CaM also mediates MAPK downregulation. MAP kinase phosphatase 1 (MKP1), which interacts with MPK3, MPK4, and MPK6 (Ulm et al., 2002), binds CaM in a Ca^2+^-dependent manner and stimulates MKP1 phosphatase activity (Lee et al., 2008). The associations between CDPKs and MAPK cascades have recently been review elsewhere (Wurzinger et al., 2011).

Much progress has been made in understanding how MAPK signaling functions in plant immunity. In *Arabidopsis*, 3 of the 60 identified MAP3Ks are involved in defense, namely MEKK1 (Asai et al., 2002), EDR1 (Frye et al., 2001), and MEKKα (del Pozo et al., 2004; Ren et al., 2008). In addition, at least 6 of the 10 identified MAP2Ks (MKK1, MKK2, MKK4, MKK5, MKK7, and MKK9) are involved in defense signaling (Asai et al., 2002; Djamei et al., 2007; Dóczi et al., 2007; Zhang et al., 2007b; Yoo et al., 2008). This situation requires tight regulation of the spatial and temporal kinase activities in order to impose specificity upon downstream signaling. To shed light on this regulation, high-throughput methods such as those used by Popescu et al. (2009) are particularly valuable and help to outline MAPK signaling cascades. While this progress may be lauded, further work needs to focus on identifying direct, *in vivo* kinase substrates and their respective phosphorylation sites. This may bring us closer to bridging the apparent gap between PRRs and MAPK cascades, and to understanding how specificity is achieved among MAPK pathways both spatially and temporally.

## Conflict of Interest Statement

The authors declare that the research was conducted in the absence of any commercial or financial relationships that could be construed as a potential conflict of interest.
